# Effect of adherence to nutrition guidelines and use of fortification of feeds on the growth of late preterm neonates

**DOI:** 10.1371/journal.pone.0357791

**Published:** 2026-09-08

**Authors:** Isabella DeStefano, Adanna Ibeku, Krystal Hunter, Melissa Micallef, Alla Kushnir

**Affiliations:** 1 Cooper Medical School of Rowan University, Camden, New Jersey, United States of America; 2 Research Institute, Cooper University Hospital, Camden, New Jersey, United States of America; 3 Department of Pediatrics, Division of Neonatology, Cooper University Hospital, Camden, New Jersey, United States of America; University of Michigan, UNITED STATES OF AMERICA

## Abstract

“Late preterm” or “LPT” neonates are generally defined as infants born between 34 0/7 and 36 6/7 weeks gestation and constitute approximately 74% of all preterm births. While much effort has been put into evaluating the nutritional needs of early preterm or low birthweight infants, there is a shortage of research on the nutritional needs of LPT infants. This work examined how adherence to current nutrition guidelines and how the use of fortification of feeds affected the growth of LPT neonates in the first year of life. A retrospective chart review was conducted of 898 neonates born between 34 0/7 and 36 6/7 weeks gestation, as identified from electronic medical records. The study site where the data was obtained was an urban hospital in New Jersey. Head circumference, weight, and length at birth, documented as measurements, percent, and z-scores, at discharge, at two months, at six months, and at twelve months post birth, were collected and evaluated. Data regarding the neonate’s feeding regimen, caloric fortification, and type of nutrition at the point of discharge were assessed. Data were analyzed using independent t-tests and Mann-Whittney U tests to assess the relationship between adherence and growth parameters. Of these participants, 50.1% were male, the mean gestational age was 34.91 (±1.19), and the mean birth weight was 2317 (±489) grams. Infants with lower birth weight (BW) were more likely to receive fortification (BW p < 0.001, BW percentile p < 0.001). Participants who followed fortification guidelines had lower birth weight, discharge weight, and weight percentile (p < 0.001), as well as lower z-scores for BW, length, and head circumference (HC) at birth and at discharge. At six and twelve months, weight, weight percentile, length, length percentile, head circumference, and head circumference percentiles were not statistically significant. The results show that adherence to nutritional guidelines recommending fortified feeding is associated with growth within the first year of life of LPT infants. By two, six, and twelve months, there were no differences in z scores in participants with fortification.

## Introduction

Late preterm neonates (LPT) are generally defined as infants born between 34 0/7 and 36 6/7 weeks gestation [1]. This subset of premature infants is surging, constituting approximately 74% of all preterm births and 8% of all births [2]. Despite efforts to reduce the incidence of preterm births, rates of LPT continue to increase by about 2% per year, rising 15% from 2014 to 2022 [3]. There are a variety of factors that contribute to LPT births. Approximately a quarter of LPT are the result of medical interventions, while 75% are spontaneous [2]. Factors contributing to late preterm births include increasing rates of multiples, fertility treatments, and medical interventions for conditions such as preterm premature rupture of membranes or intrauterine growth restriction [1]. Other causes of late preterm births include labor induction, cesarean delivery or other maternal comorbidities, such as gestational diabetes, chronic hypertension, renal complications, cardiac disease, and autoimmune conditions [2].

LPT birth often presents challenges for the newborn, due to their shortened development in utero. The delivery of nutrients, including folate, iron, magnesium, calcium and zinc during the third trimester of pregnancy is disrupted and, as a result, the LPT neonate can suffer from underdevelopment of essential organs, including the brain and intestine [4]. LPT neonates are also susceptible to developing hypoglycemia, feeding difficulties, dehydration, postnatal growth restriction, respiratory distress, jaundice, and infection [5].

One area of particular concern is optimizing nutritional management for LPT neonates. Growth in the LPT infant is a problem because currently there is not a consensus on best practices for LPT nutrition, resulting in a large variation of care [6]. Complicating this challenge is the fact that many LPT neonates are cared for outside of the neonatal intensive care unit. Ascertaining proper nutritional and caloric requirements for this population is critical because growth failure is common; and the goal is to catch up to a healthy full-term infant of the same gestational age [7]. Inadequate nutrition post-discharge can also lead to failure to thrive or metabolic disorders that could have long-term impact into adulthood [8]. Thus, fortification of feeds of LPT neonates include consideration of the calories, vitamins, minerals, and nutrients necessary to support growth and development [4].

When considering the optimal feeding regimen for LPT infants, it is well-established that human milk from the LPT neonate’s own mother is the preferred nutritional source [6]. However, human milk is not always available, or in sufficient quantities, thus necessitating the use of formula as either a primary or supplemental source of nutrition [9].

Preterm infant formulas are used when human milk is not available, insufficient, unable to be tolerated, or, as a method of increasing caloric intake as a method of fortification. Typically, the macronutrient content of infant formula is the same as human milk, at 20kcal/oz [8]. In many NICU’s, standardized feeding programs are instituted in an effort to optimize nutrition and promote growth of premature infants [10]. In many of those cases, clinicians may adjust the feeding regimen to increase calories up to 22kcal/oz or 24 kcal/oz [11].

While much effort has been put into developing feeding programs to meet the needs of early preterm or low birthweight infants, there is a shortage of research on optimal nutritional strategies benefitting LPT infants. Thus, determining the nutritional needs of this population poses a challenge to clinicians. One approach to optimizing the nutritional needs of LPT neonates involves adhering to a fortified feeding regimen of 22 kcal/oz or 24 kcal/oz per feed. Adherence to current nutrition guidelines and the fortification of feeds may affect the growth of late preterm neonates [12].

This study is significant since consensus is lacking on guidelines to optimizing nutrition for late preterm neonates in the first year of life. The objective of this study was to examine how implementation of a fortified feeding regimen comprised of 22 kcal/oz or 24 kcal/oz per feed affects the growth of LPT neonates in the first year of life.

## Methodology

### Study population

This retrospective cohort study was approved by the hospital Institutional Review Board under IRB #23–138 with the determination that it was exempt under IRB exemption category 4(iii) with a HIPAA Waiver for participant informed consent and authorization for release of protected health information (PHI), in accordance with the Health Insurance Portability and Accountability Act (HIPAA). Participant lists were generated from the electronic medical record (EPIC) data set at an urban hospital in New Jersey, USA. The institutional data analysts applied the inclusion and exclusion criteria to generate a list of medical records for chart review. Eligible participants were identified as late preterm neonates born between 34 0/7 and 36 6/7 weeks gestation, from 1/2013–12/2022. Eligible participants were excluded from the study if the electronic medical records indicated a significant congenital and/or genetic anomaly or if the composition of the participant’s feeds or caloric intake of the feeds was incomplete or missing.

### Sampling strategy

898 subjects were analyzed to allow for a broad cross-section of subjects. This sample size also allowed for adequate data points for analysis. Convenience sampling was utilized since all participants were born at the same hospital. The data collectors were trained, mentored and supervised by one of the coauthors, a neonatologist. Training included careful review of the variables, procedures to access the electronic medical record, and the data abstraction form. The data were accessed between 7/14/2023–12/26/2023 for research purposes. The non-anonymized participant data, including gestational age, composition of feeding, quantity per feed, head circumference, length, and weight measurements, were extracted from the electronic medical record and kept in a password-protected file. All participant information was then anonymized for analysis.

There were variations in the sample size for different variables due to participant attrition. Variations in the sample sizes for different variables occurred due to adoption of pairwise deletion for the dataset.

### Explanatory variables

The objectives of the project were to obtain data regarding the LPT neonates’ feeding regimens and growth at various points over the twelve months following birth. Growth was evaluated using approximate instrument derived measurements of weight, length and head circumference, as well as z scores for all parameters. Z-scores were defined in the study as age and gender based standardized scores quantified by the number of deviations below or above the median, as calculated with reference to the Fenton Growth Charts, and accessed via the EPIC electronic record system. This was done at the time of the subject’s discharge, two months, six months, and twelve months post birth. The z scores in this study were typical for neonates of the population at this hospital system. Demographic data were also collected on all subjects and their mothers. Demographic data included race, insurance, maternal employment, and maternal marital status.

Data related to feeding regimen of each subject included the type of feeding (formula, maternal human milk, donor human milk, combination formula and human milk, or other) and caloric formulation while in the hospital and at discharge from the hospital. For purposes of this study, human milk was assumed to have a macronutrient content of 20kcal/oz, standard infant formula had a macronutrient content of 20 kcal/oz, fortified infant formula had a macronutrient content of 22 kcal/oz or above, and a feeding regimen combining human milk and formula had a macronutrient content of 20 kcal/oz or greater. It should be noted that human milk was assumed to have a macronutrient content of 20kcal/oz, despite research that shows that a wide variation in energy and protein in human milk exists and usually decreases over time [13].

### Outcome measures

In this hospital, the standard practice in the neonatal intensive care unit (NICU) was to place all preterm neonates on 22 or 24 kcal/oz nutrition. This was not always done in the mother-infant unit (MIU), where general pediatricians from various practices were responsible for managing the infants and given the lack of standardized guidelines related to LPT nutrition. For the purposes of this study, “adherence” was defined as providing feeds of 22 kcal/oz or greater at the time of discharge and growth was assessed during the first 12 months of life. “Adherence” was determined at the point of discharge with the intent to continue to adhere, post discharge. Participants were categorized as adhering if the documented caloric content of feeds met or exceeded 22 kcal/oz at the time of discharge. Limitations of this study were participant attrition, the inability to fully determine post-discharge feeding practices, and the exclusion of participants who were lost to follow up from analysis. The participants with missing data were eliminated from the analysis via pairwise deletion.

Data related to the growth of each subject were recorded, including head circumference (HC), weight, and length at birth (documented as measurements, percentile, and z-scores), at discharge, two months, six months, and at twelve months post birth.

### Statistical analysis

For all continuous variables, we ran a test of skewness to determine the normality of the data. For those variables that has a level of skewness that fell between −1.5 to 1.5 that indicated that that data had a normal distribution without minimal level of skew. For these variables, an independent t test was run. If the value of skewness was less than −1.5 or greater than 1.5, that indicated that there was a high level of skew. For these data elements, Mann Whitney U tests were run. The statistical software used was SPSS 27 (IBM, Armonk, NY). In order to examine the test of skewness for each continuous data element, please review S1 Table. While it is recognized that not all of the data fall into the same category, it was decided to report non-parametric results for consistency in data reporting and to eliminate confusion. Homogeneity of variance was assessed by using the Levene’s test.

The size of the statistical groups varied, based on data availability and participant follow up. If the data were available within the participant’s electronic medical record, they were included in the analysis. The availability of the data for each metric is delineated in the Supplemental Materials. All relevant data are within this work and its Supporting Materials. Measurements, percentiles, and z-scores were analyzed comparing participants who received a fortified feeding regimen of ≥22 kcal/oz (adherent) versus those that received breast milk or formula that was not fortified (non-adherent). Statistical significance was set at p < 0.05. The unadjusted significance level is p < 0.05 and the Bonferroni corrected significance level is p < 0.001. Results that are significant when unadjusted and not significant when Bonferroni adjusted are denoted with a star in the tables.

The multivariate statistical analysis results are summarized in the tables. Linear regression was the appropriate statistical method for the multivariable analysis. Effect sizes are also found in the tables. Effect sizes were calculated for the data analyzed using Mann Whitney U tests and independent t-tests. The effect sizes were calculated using 95% confidence intervals for multivariable models. This study utilized an intention to treat design, with all participants assigned to either the adhering or non-adhering category at the point of discharge. All participants remained in their assigned category as growth was tracked through the first year of life. A post hoc analysis was completed for items that were not significant (S3 Table).

## Results

This study was conducted at a tertiary medical center and involved 898 participants. The mean gestational age was 34.91 (± 1.19) weeks, and mean birth weight was 2317 (± 489) grams. Mann Whitney U tests were used for analyzing the z-scores. The post hoc analysis indicates that the sample required a power of 80% (S3 Table).

Of the 898 subjects, 589 received a fortified feeding regimen of 22 kcal/oz or 24 kcal/oz per feed. The mean birthweight (BW) among adherent neonates was 2177.35 grams (± 439.63). Of 213 infants who were non-adherent, the mean BW was 2614.85 grams (±441.63)(p < 0.001, effect size of −0.994). BW percentiles of 587 neonates who adhered were significantly lower, as compared to 207 participants who did not adhere (33.89 ± 26.90 vs 44.38 ± 28.35, p < 0.001, effect size of −0.384) (Table 1).

**Table 1:** Participants’ demographics of gender, race, insurance, maternal employment, and maternal marital status.

Variables	N (%)
Gender	Male	450 (50.1)
	Female	448 (49.8)
Race	White	373 (41.5)
	Black	296 (33.0)
	Hispanic	143 (15.9)
	Asian	27 (3.01)
	Other	21 (2.34)
Insurance	Medicaid	294 (32.7)
	Medicare	21 (2.34)
	Private	438 (48.8)
	Other	18 (2.00)
	None	52 (5.79)
Maternal Employment	Employed	215 (23.9)
	Unemployed	683 (76.1)
Maternal Marital Status	Married	235 (26.3)
	Single	570 (63.4)
	Other	23 (2.06)

At birth, the mean length, length percentile, HC and HC percentiles of adherent neonates were significantly lower than those who were not. At discharge, all of the growth parameters were statistically significantly lower in infants who were fortified prior to discharge, as compared to those who did not receive fortified feeds (Table 2).

**Table 2:** Mean growth parameters from birth to twelve months.

Independent T test										
Adherence = “Yes”			Adherence = “No”					95% Confidence Interval of Effect Size	
N	Mean	StDev	N	Mean	StDev	P-Value	Effect Sizes	Lower Limit	Upper Limit
**BW**	**589**	**2177.3514**	**439.62869**	**213**	**2614.8545**	**441.63328**	**<0.001**	**−0.994**	**−1.158**	**−0.83**
**BW percentile**	**587**	**33.8881**	**26.90381**	**207**	**44.3771**	**28.34668**	**<0.001**	**−0.384**	**−0.544**	**−0.225**
**Length at birth**	**588**	**44.9486**	**2.86194**	**213**	**47.0718**	**2.77301**	**<0.001**	**−0.748**	**−0.909**	**−0.587**
**Length Percentile at birth**	**582**	**44.0327**	**30.10106**	**208**	**53.4234**	**29.49144**	**<0.001**	**−0.314**	**−0.473**	**−0.154**
**Head circumference at birth**	**589**	**30.8819**	**2.19072**	**213**	**32.2601**	**1.99285**	**<0.001**	**−0.644**	**−0.804**	**−0.484**
**Head circumference percentile at birth**	**581**	**37.5218**	**29.45151**	**208**	**44.9543**	**31.66306**	0.003*	**−0.247**	**−0.406**	**−0.088**
**Weight at discharge**	**551**	**2432.9926**	**549.09578**	**175**	**2753.1371**	**636.26496**	**<0.001**	**−0.56**	**−0.733**	**−0.388**
**Weight percentile at discharge**	**549**	**20.9827**	**21.57027**	**171**	**32.826**	**27.79616**	**<0.001**	**−0.511**	**−0.684**	**−0.337**
**Length at discharge**	**545**	**46.071**	**4.05604**	**174**	**47.6239**	**3.26776**	**<0.001**	**−0.4**	**−0.572**	**−0.228**
**Length Percentile at discharge**	**543**	**31.7733**	**26.90142**	**170**	**39.6298**	**29.9572**	0.002*	**−0.284**	**−0.457**	**−0.111**
**Head circumference at discharge**	**548**	**32.0393**	**3.0879**	**174**	**33.1149**	**1.98153**	**<0.001**	**−0.376**	**−0.547**	**−0.204**
**Head circumference percentile at discharge**	**544**	**33.118**	**27.06345**	**170**	**43.138**	**29.14409**	**<0.001**	**−0.363**	**−0.537**	**−0.19**
**Weight at 2 months**	**295**	**4363.1846**	**1033.83875**	**94**	**4597.7128**	**930.38707**	**0.051**	**−0.232**	**−0.465**	**0.001**
**Weight percentile at 2 months**	**295**	**43.1729**	**31.10551**	**93**	**44.168**	**29.9333**	**0.786**	**−0.032**	**−0.265**	**0.201**
**Length at 2 months**	**371**	**67.8833**	**293.9898**	**104**	**53.7038**	**3.09051**	**0.623**	**0.055**	**−0.163**	**0.272**
**Length Percentile at 2 months**	**360**	**20.9715**	**25.79741**	**101**	**25.3579**	**25.23376**	**0.13**	**−0.171**	**−0.392**	**0.05**
**Head circumference at 2 months**	**364**	**37.1261**	**1.70388**	**101**	**37.7129**	**1.5863**	0.002*	**−0.349**	**−0.571**	**−0.128**
**Head circumference percentile at 2 months**	**364**	**36.6237**	**31.77761**	**101**	**42.2032**	**32.06368**	**0.12**	**−0.175**	**−0.396**	**0.046**
**Weight at 6 months**	**242**	**7280.314**	**1177.5337**	**76**	**7268.0107**	**1310.25556**	**0.938**	**0.01**	**−0.248**	**0.268**
**Weight percentile at 6 months**	**242**	**58.6049**	**33.50198**	**76**	**58.3193**	**29.73804**	**0.947**	**0.009**	**−0.249**	**0.266**
**Length at 6 months**	**241**	**95.1029**	**484.16279**	**66**	**65.2788**	**2.71171**	**0.618**	**0.069**	**−0.203**	**0.342**
**Length Percentile at 6 months**	**239**	**26.9749**	**25.21308**	**66**	**35.4836**	**27.70314**	0.018*	**−0.33**	**−0.604**	**−0.056**
**Head circumference at 6 months**	**236**	**42.7126**	**2.98336**	**64**	**42.668**	**1.51111**	**0.908**	**0.016**	**−0.26**	**0.293**
**Head circumference percentile at 6 months**	**235**	**39.4649**	**30.54184**	**64**	**39.1898**	**27.98576**	**0.948**	**0.009**	**−0.267**	**0.286**
**Weight at 12 months**	**289**	**9421.1422**	**1699.74239**	**84**	**9354.5973**	**1616.88392**	**0.75**	**0.04**	**−0.203**	**0.283**
**Weight percentile at 12 months**	**286**	**58.0946**	**32.6818**	**84**	**57.5707**	**32.92988**	**0.897**	**0.016**	**−0.227**	**0.259**
**Length at 12 months**	**265**	**73.1272**	**3.48514**	**78**	**73.5205**	**3.0122**	**0.368**	**−0.116**	**−0.369**	**0.136**
**Length Percentile at 12 months**	**263**	**35.1995**	**28.09278**	**79**	**35.72**	**27.62917**	**0.885**	**−0.019**	**−0.27**	**0.233**
**Head circumference at 12 months**	**259**	**45.9548**	**2.45999**	**77**	**45.7494**	**1.70214**	**0.494**	**0.089**	**−0.166**	**0.343**
**Head circumference percentile at 12 months**	**255**	**56.8042**	**31.34438**	**77**	**49.7536**	**30.5722**	**0.083**	**0.226**	**−0.029**	**0.481**

At two months post birth, there was no statistical difference in neonates for mean weight, weight percentile, length, length percentile, head circumference, or head circumference percentile. Similarly, neonates who adhered to fortification had lower mean length and length percentiles at two months, as compared to their peers. However, the mean head circumference for adhering infants was statistically greater at 37.13 ± 1.70, as compared to 37.71 ± 1.59, p = 0.002, effect size of −0.349) (Table 3).

**Table 3:** Median Z scores for weight, length and head circumference from birth to twelve months using Mann Whitney U Test.

Mann Whitney U Test								
Adherence = “No”		Adherence = “Yes”				95% Confidence Interval of Effect Size	
N	Median (25th percentile, 75th percentile)	N	Median (25th percentile, 75th percentile)	P-Value	Effect Size	Lower Limit	Upper Limit
**BW Z score**	**208**	**−0.215 (−0.7325, 0.4225)**	**587**	**−0.56 (−1.31, 0.12)**	**<0.001**	**−0.182**	**−0.251**	**−0.112**
**Length Z score at birth**	**208**	**0.15 (−0.565, 0.7725)**	**585**	**−0.16 (−0.96, 0.54)**	**<0.001**	**−0.133**	**−0.203**	**−0.062**
**Head circumference Z score at birth**	**208**	**−0.175 (−0.98, 0.76)**	**586**	**−0.425 (−1.2425, 0.315)**	0.004*	**−0.102**	**−0.172**	**−0.03**
**Weight Z score at discharge**	**171**	**−0.63 (−1.39, 0.01)**	**550**	**−1.1 (−1.68, −0.4775)**	**<0.001**	**−0.186**	**−0.258**	**−0.113**
**Length Z score at discharge**	**170**	**−0.375 (−1.1775, 0.32)**	**544**	**−0.68 (−1.4375, 0.015)**	0.002*	**−0.115**	**−0.189**	**−0.04**
**Head circumference Z score at discharge**	**171**	**−0.23 (−0.92, 0.49)**	**547**	**−0.59 (−1.31, 0.11)**	**<0.001**	**−0.164**	**−0.236**	**−0.089**
**Weight Z score at 2 months**	**93**	**−0.17 (−0.905, 0.535)**	**295**	**−0.16 (−1.2, 0.49)**	**0.477**	**−0.036**	**−0.138**	**0.067**
**Length Z score at 2 months**	**104**	**−1.03 (−1.72, −0.255)**	**370**	**−1.45 (−2.3325, −0.41)**	0.014*	**−0.113**	**−0.203**	**−0.02**
**Head circumference Z score at 2 months**	**101**	**−0.34 (−1.065, 0.685)**	**364**	**−0.54 (−1.4775, 0.4)**	**0.089**	**−0.079**	**−0.171**	**0.015**
**Weight Z score at 6 months**	**76**	**0.155 (−0.425, 1.1025)**	**242**	**0.405 (−0.63, 1.21)**	**0.631**	**0.027**	**−0.087**	**0.14**
**Length Z score at 6 months**	**66**	**−0.5 (−1.26, 0.155)**	**241**	**−0.94 (−1.74, −0.08)**	0.017*	**−0.137**	**−0.248**	**−0.022**
**Head circumference Z score at 6 months**	**64**	**−0.265 (−1.0275, 0.2275)**	**236**	**−0.375 (−1.1925, 0.43)**	**0.994**	**0**	**−0.116**	**0.117**
**Weight Z score at 1 year**	**84**	**0.305 (−0.69, 1.2725)**	**288**	**0.32 (−0.4675, 1.1425)**	**0.798**	**0.013**	**−0.091**	**0.118**
**Length Z score at 1 year**	**78**	**−0.575 (−1.1725, 0.2625)**	**264**	**−0.585 (−1.155, 0.1275)**	**0.662**	**−0.024**	**−0.133**	**0.086**
**Head circumference Z score at 1 year**	**77**	**−0.03 (−0.685, 0.6)**	**260**	**0.305 (−0.58, 1.135)**	**0.066**	**0.100**	**−0.010**	**0.208**

**Fig 1:**
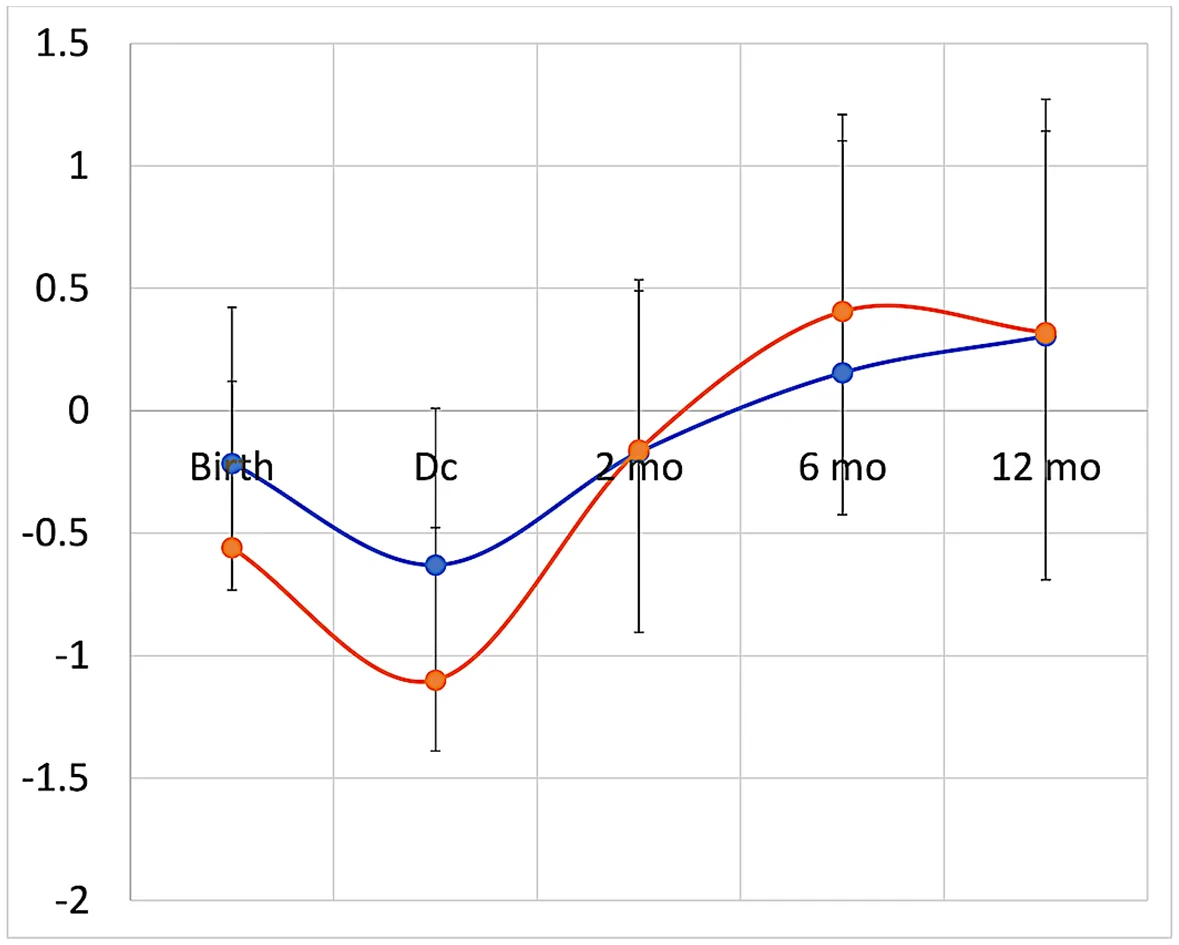
Comparison of Median Weight Z-Scores in Late Preterm Neonates who Received Fortification at Discharge and Those Who Did Not.

The adhering infants were able to surpass the weight of non-adhering infants, as demonstrated by an increase in median z-scores at six months, as compared to two months, 0.565 versus 0.325. This gap narrowed further when the infants were twelve months old, with median weight z-scores of 0.32 (adherent group) versus 0.305 (non-adherent) (Fig 1). The infants who received fortification were able to attain weight similar to the infants who were not fortified by two months, with a median score −0.16 vs −0.17.

**Fig 2:**
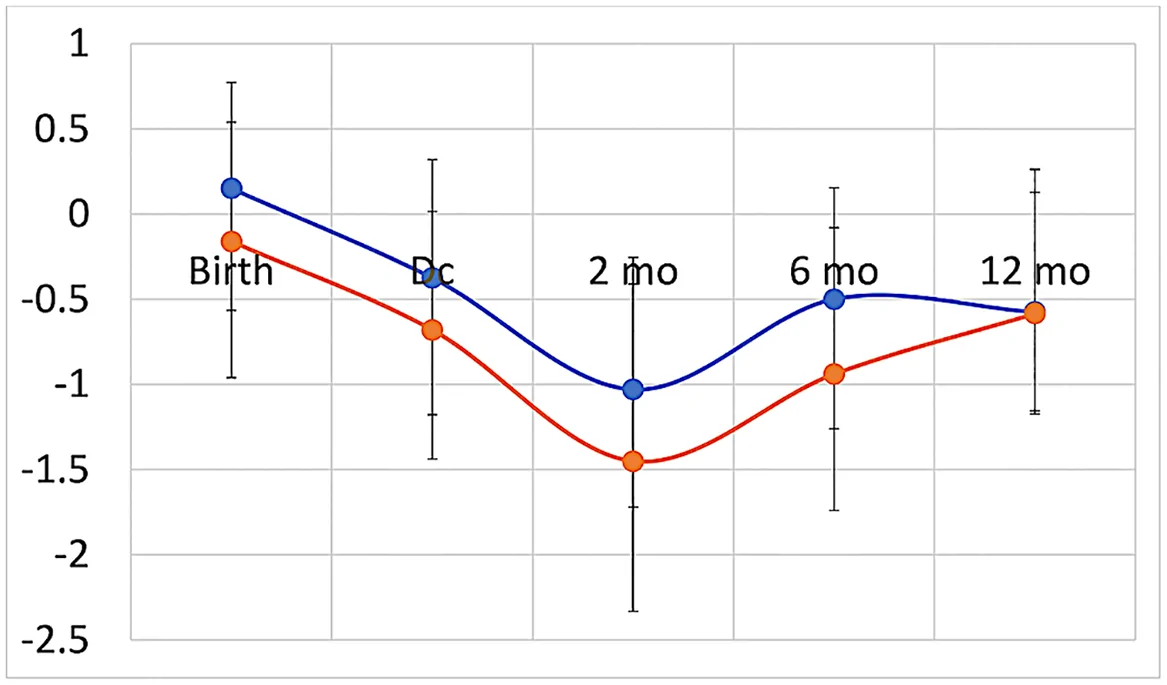
Comparison of Median Length Z-scores in Late Preterm Neonates who Received Fortification at Discharge and Those Who Did Not.

**Fig 3:**
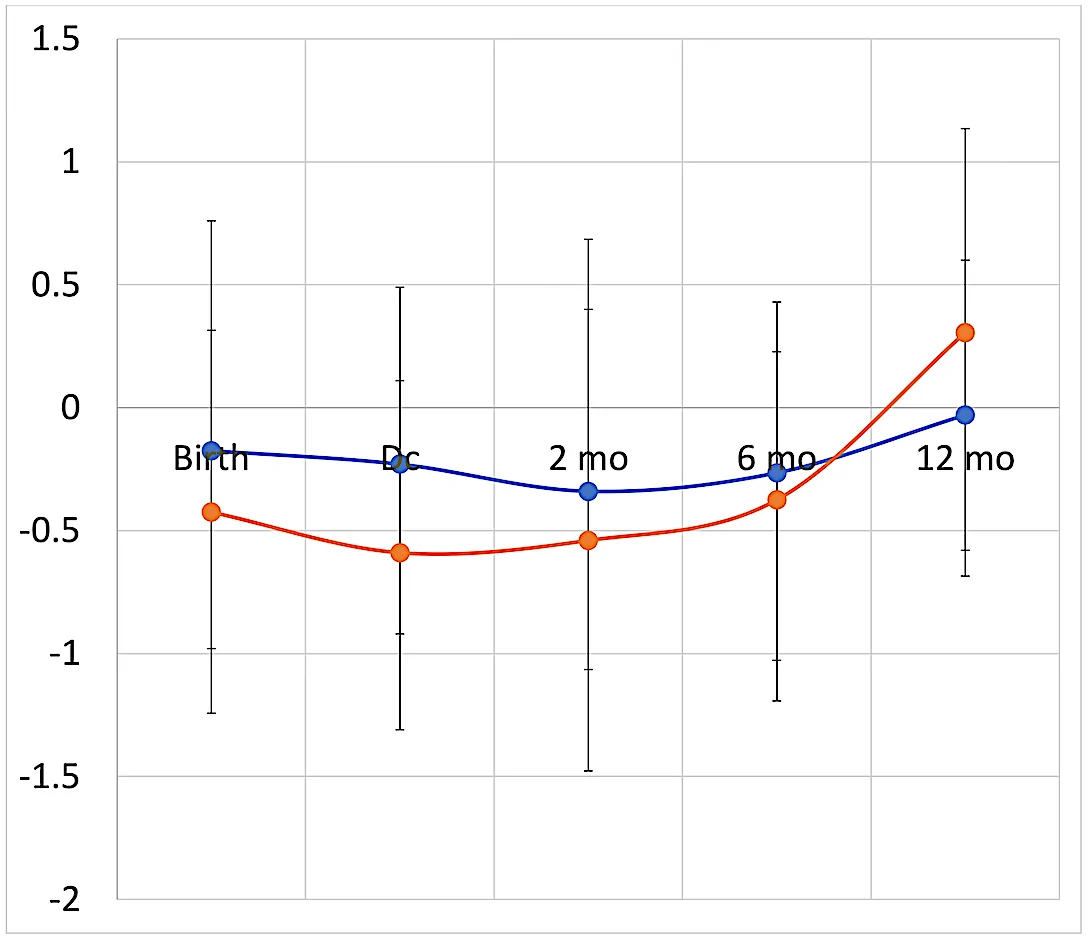
Comparison of Median Head Circumference Z-Scores in Late Preterm Neonates who Received Fortification at Discharge and Those Who Did Not.

The neonates who received fortification had lower lengths from birth until six months, compared to neonates who did not receive fortification. However, the neonates who received fortification had similar length z-scores by twelve months of age, as shown by the median z-score −0.585 (adherent) and 0.575 (non-adherent) (Fig 2). Neonates who received fortification had lower median head circumference z-scores at birth until 6 months. The head circumference z-scores of neonates who received fortified feeds at birth were significantly higher compared to neonates who did not adhere by twelve months of age, as demonstrated by the median z-score of 0.305 versus −0.03 (Fig 3). The change in median z-score was an increase of 0.68 for adhering infants.

At twelve months post birth, neonates who adhered to a fortified feeding regimen were able to catch up to the growth of their non-adhering peers, despite starting at lower growth parameters. The adhering neonate mean weight at twelve months was 9421.14 ± 1699.74, as compared to 9354.60 ± 1616.88 p = 0.750 effect size of 0.040 for non-adhering infants. The mean length at twelve months for adhering infants was 73.13 ± 3.49, as compared to 73.52 ± 3.01 p = 0.368 effect size of −0.116. The mean head circumference for neonates who adhered was 45.95 ± 2.46, as compared to 45.75 ± 1.70 p = 0.494 effect size of 0.089 (Table 4).

**Table 4:** Growth parameters at twelve months, as compared to constants using linear regression.

Outcome = Weight at 1 Yr			Outcome = Weight at 1 Yr Percentile			Outcome = Length at 1 Yr			Outcome = Length at Yr Percentile			Outcome = Head Circum 1 Yr			Outcome = Head Circum Percentile at 1Yr		
B	t	P-Value	B	t	P-Value	B	t	P-Value	B	t	P-Value	B	t	P-Value	B	t	P-Value
**(Constant)**		**2.946**	0.003*		**−2.157**	0.032*		**19.036**	**<0.001**		**−2.467**	0.014*		**20.255**	**<0.001**		**−1.607**	**0.109**
**BW**	**0.941**	**2.911**	0.004*	**1.197**	**3.448**	**< 0.001**	**1.513**	**4.511**	**<0.001**	**1.187**	**3.413**	**< 0.001**	**1.032**	**3.352**	**<0.001**	**1.092**	**3.494**	0.001*
**GA = 34.0**	**−0.110**	**−0.377**	**0.706**	**0.473**	**1.363**	**0.174**	**−0.003**	**−0.011**	**0.991**	**0.176**	**0.563**	**0.574**	**0.013**	**0.168**	**0.867**	**0.051**	**0.667**	**0.505**
**GA = 35.0**	**0.102**	**1.531**	**0.127**	**0.164**	**0.535**	**0.593**	**0.058**	**0.829**	**0.408**	**0.017**	**0.237**	**0.813**	**0.042**	**0.577**	**0.564**	**0.011**	**0.148**	**0.882**
**Formula**	**0.198**	**1.517**	**0.130**	**0.201**	**1.549**	**0.122**	**0.140**	**1.004**	**0.316**	**0.170**	**1.175**	**0.241**	**0.152**	**1.019**	**0.309**	**0.123**	**0.823**	**0.411**
**Breast**	**0.066**	**0.882**	**0.379**	**0.001**	**0.016**	**0.987**	**0.115**	**1.419**	**0.157**	**0.079**	**0.941**	**0.347**	**0.160**	**1.855**	**0.065**	**0.102**	**1.167**	**0.244**
**Vaginal Delivery**	**−0.005**	**−0.091**	**0.928**	**−0.020**	**−0.362**	**0.718**	**−0.043**	**−0.736**	**0.462**	**−0.039**	**−0.633**	**0.527**	**−0.021**	**−0.337**	**0.736**	**−0.079**	**−1.259**	**0.209**
**Cal_22–24**	**−0.155**	**−1.471**	**0.142**	**−0.223**	**−2.124**	0.034*	**−0.097**	**−0.874**	**0.383**	**−0.135**	**−1.176**	**0.241**	**−0.008**	**−0.071**	**0.944**	**−0.123**	**−1.039**	**0.300**
**INT_GA34_BW**	**0.296**	**1.054**	**0.293**	**−0.260**	**−0.792**	**0.429**	**0.136**	**0.474**	**0.636**	**−0.027**	**−0.091**	**0.927**						
**BWsq**	**−0.691**	**−2.245**	0.025*	**−0.840**	**−2.696**	0.007*	**−1.204**	**−3.793**	**<0.001**	**−0.924**	**−2.809**	0.005*	**−0.864**	**−2.810**	0.005*	**−0.918**	**−2.946**	0.003*
**INT_GA35_BW**				**−0.033**	**−0.111**	**0.911**												
**Rsq = 0.127**				**Rsq = 0.155**			**Rsq = 0.169**			**Rsq = 0.109**			**Rsq = 0.069**			**Rsq = 0.068**		

*B= Standardized Coefficients Beta

At twelve months, there is a curvilinear relationship between birth weight and weight, as compared to weight, weight percentiles, length, length percentile, head circumference, and head circumference percentile. There is no statistically significant change in growth based on gestational age of 34 or 35 weeks, type of feeding, or type of birth delivery. The infants who adhered to fortified feeding had a statistically significant lower weight percentile (Beta = −0.223, t = −2.124, p = 0.034).

## Discussion

The hypothesis of the current study was that among late preterm neonates, adherence to a fortified feeding regimen of 22 kcal/oz or 24 kcal/oz per feed at discharge and after would improve growth in the first twelve months of life. The infants who adhered to fortification regimens had lower head circumferences (HC), length, and weights at birth. However, by twelve months post birth, those neonates were able to reach or exceed all growth parameters (weight, length and HC) exhibited by those who did not receive fortification.

These findings suggest that by two months, adhering LPT neonates had statistically significant growth in head circumference and weight. After two months of life, the adhering infants surpassed the weight of non-adhering infants, and by twelve months, they caught up in median length. These findings support the implementation of a fortified feeding regimen of 22 kcal/oz or 24 kcal/oz per feed for LPT neonates in order to optimize growth.

Guidance on nutritional practices for late and moderately late preterm infants is sparse and lacking in consensus; some researchers have identified clinical practices for this population [5]. Muelbert’s work confirms that there is a lack of studies for this subset of infants [1]. LPT infants tend to experience feeding difficulties, compounding the challenge of optimizing nutrition for this population [14]. As LPT births increase, there is a greater need for developing nutritional best practices so as to minimize variations in clinical practice [1]. Muelbert’s research found that while rapid growth velocity may be desired for neurocognitive function, it could be detrimental to later metabolic health. Additionally, it should be noted the current study acknowledges Muelbert’s observation that while breastfeeding is beneficial for preterm infants, there are special logistical challenges in implementation for this population [1].

As Zhang, et al. explains, it is critical that LPT infants optimize postnatal growth to improve their survival, neurodevelopment, and lower metabolic risks [15]. Zhang’s research comports with the current study’s observation that there is not a consensus on the most suitable growth charts to monitor and evaluate the postnatal growth of this population. Contradictory to Muelbert [1], Zhang did not find any neonatal complications in analysis of growth velocities. Zhang points out that the Fenton-fetal infant reference has been widely used in evaluating postnatal growth of preterm infants, but the growth trajectory/velocity of LPT infants has not been widely studied [15]. The results here correspond with that observation and also utilized the Fenton growth curve infant reference.

Similar to our study, Lin et al. found that nutritional supplementation may improve early growth for infants born prematurely or small for gestational age [12]. Unlike the current study, Lin studied the effects of long-term growth, and evaluated effect of gender. The authors concluded preterm children born between 29–32 weeks who received supplements had lower BMI as toddlers and in childhood than un-supplemented children, but those born extremely or moderate to late preterm did not [12].

The challenges of ascertaining optimized nutrition for LPT infants is highlighted in a large cohort study by Santos [16]. That cohort was followed for twenty-four months, assessing growth outcomes in Brazil. The LPT infants were compared to full-term infants under a comprehensive set of criteria than the current study, including maternal characteristics, child characteristics (at twelve and twenty-four months), whether the infant was underweight, nutritional source and duration, and health concerns including wheezing, pneumonia, diarrhea, hospitalization, stunting, and/or wasting [16]. Santos found that while LPT infants eventually gained as much weight as the full-term infants, they generally experienced stunting and were underweight for their age. The infants also had higher hospitalization rates [16]. Their findings highlight the importance of optimizing nutrition for the LPT neonates since they have a 2.57 greater chance of being underweight at twelve months than a term infant and a 3.36 higher chance of being underweight at two years of age [16]. Unlike our study, in Santos’ study, mothers of LPT infants also reported shorter breastfeeding duration than those who had term infants [16].

The current study has several strengths. It had a large sample size of approximately 900 subjects, both male and female, born over multiple years, as well as singletons and multiple births. We also evaluated neonates who required NICU stay and those that did not, as well as those who received human milk, formula, or combined nutrition. The goal of this work was to evaluate growth outcomes in an effort to help standardize clinical recommendations for optimizing nutrition for this subset of neonates.

Some of the limitations of the current study include it not being randomized or blinded, and retrospective data evaluation. The length of time an infant adhered to a fortified feeding regimen was not clearly documented beyond the point of discharge, due to the difficulty in evaluating dietary intake in the electronic medical record post-discharge. Limitations of this study were participant attrition, the inability to fully determine post-discharge feeding practices, and the exclusion of participants who were lost to follow up from analysis. Participants with missing data were excluded from the analysis via a pairwise deletion. Thus, the clinical outcomes are short-term. This work may not be generalizable to all LPT infants. Since this study did not verify fortification post-discharge, it could result in limitations for causal inference.

This study demonstrates the importance of creating standardized guidelines regarding the need for adherence to fortified nutrition for LPT neonates in order to optimize their growth. Even though LPT infants constitute a minority of all infants, there is a lack of research on nutritional best practices for this cohort. From the clinical perspective, development of standardized procedures to fortify nutrition for this vulnerable population can positively affect growth within the first twelve months of life. Future studies can expand on this work to monitor LPT infant growth beyond twelve months of age to assess trends in nutrition and development and refine the metabolic concerns that may be associated with catch-up growth. This research also underscores the importance of education in facilitating adherence to clinical nutritional guidelines.

## Conclusion

In this study, LPT neonates with lower BW, discharge weight, and discharge weight percentile were more likely to receive fortification at the time of discharge. Participants who were fortified had statistically lower z-scores for BW, length, and HC at birth and at discharge. By two, six, and twelve months, there were no differences in weight, length and HC z scores in participants with fortification. The results demonstrate that adherence to nutritional guidelines recommending fortified feeding was linked to growth within the first year of life of late premature neonates. These findings demonstrate that catch up growth by six months for LPT neonates with lower weight, length, and head circumference at birth was associated with adhering to a fortified feeding regimen.

## Supporting information

S1 Table Skewness Results. (PDF)

S2 Table Shapiro Wilk Results. (PDF)

S3 Table Post Hoc Analysis. (PDF)
