# Supplementary material for: Effect of adherence to nutrition guidelines and use of fortification of feeds on the growth of late preterm neonates
Source: PLoS One. 2026 Sep 8;21(9):e0357791. doi: 10.1371/journal.pone.0357791 (PMC13552749; doi:10.1371/journal.pone.0357791)
Supplement: Supplementary file 1 [file pone.0357791.s001.pdf]

	N			
	Valid	Missing	Skewness	Std. error of skewness
BW Z score	877	21	14.886	0.083
Length Z score at birth	874	24	14.017	0.083
Head circumference Z score at birth	876	22	-7.671	0.083
Weight Z score at discharge	793	105	12.139	0.087
Length Z score at discharge	785	113	6.102	0.087
Head circumference Z score at discharge	790	108	13.758	0.087
Weight Z score at 2 months	411	487	-0.0329	0.12
Length Z score at 2 months	504	394	-0.521	0.109
Head circumference Z score at 2 months	495	403	1.488	0.11
Weight Z score at 6 months	337	561	-0.237	0.133
Length Z score at 6 months	330	568	-0.92	0.134
Head circumference Z score at 6 months	320	578	10.472	0.136
Weight Z score at 1 year	400	498	0.312	0.122
CDC Weight Z score at 1 year	411	487	0.13	0.12
Length Z score at 1 year	364	534	-0.212	0.128
