# Supplementary material for: Effect of adherence to nutrition guidelines and use of fortification of feeds on the growth of late preterm neonates
Source: PLoS One. 2026 Sep 8;21(9):e0357791. doi: 10.1371/journal.pone.0357791 (PMC13552749; doi:10.1371/journal.pone.0357791)
Supplement: Supplementary file 2 [file pone.0357791.s002.pdf]

	Shapiro Wilk		
	Statistic	df	Sig.
Weight Z score at 2 months	0.97	144	0.003
Length Z score at 2 months	0.992	144	0.573
Weight Z score at 6 months	0.98	144	0.031
Length Z score at 6 months	0.986	144	0.161
Weight Z score at 1 year	0.99	144	0.375
CDC Weight Z score at 1 year	0.987	144	0.192
Length Z score at 1 year	0.986	144	0.162
