# Supplementary material for: Effect of adherence to nutrition guidelines and use of fortification of feeds on the growth of late preterm neonates
Source: PLoS One. 2026 Sep 8;21(9):e0357791. doi: 10.1371/journal.pone.0357791 (PMC13552749; doi:10.1371/journal.pone.0357791)
Supplement: Supplementary file 3 [file pone.0357791.s003.pdf]

Post Hoc analysis	Adherence vs. Nonadherence
Weight z-score at 2 months	1860
Weight z-score at 6 months	>100,000
Weight z-score at 12 months	>50,000
Head circumference z-score at 2 months	1398
Head circumference z-score at 6 months	5738
Head circumference z-score at 12 months	2418
Length z-score at 12 months	>10,000
